# Homicide-Suicide Partners: A Simulation of Injuries

**DOI:** 10.7759/cureus.39976

**Published:** 2023-06-05

**Authors:** Shailesh M Raut, Swapnil P Akhade, Pankaj S Ghormade, Tamilarasan Murugesan, Krishnadutt H Chavali

**Affiliations:** 1 Forensic Medicine & Toxicology, All India Institute of Medical Sciences, Raipur, Raipur, IND

**Keywords:** simulation injuries, homicide-suicide, intimate partners, dyadic death, facsimile injuries

## Abstract

Death by homicide-suicide or dyadic death is rare, with the nature of the death varying from case to case. The perpetrators are usually males and most often use weapons available in their vicinity to commit a crime. This case presents an instance of dyadic death using multiple methods to kill the intimate partner, followed by mirror imaging of similar injuries on himself and finally committing suicide by hanging. This case depicts a rare case of murder-suicide in which both victims and perpetrators died by different methods but a mirroring pattern of fatal injuries was observed on each intimate partner. The non-fatal injury for one was a facsimile of a fatal injury on a corresponding intimate partner.

## Introduction

The term homicide-suicide refers to when the perpetrator of a homicide takes his or her own life after killing the victim [1,2]. Several terms have been used to describe it, including murder-suicide, dyadic death, and homicide following suicide, with special mention of such instances in the literature of the Chinese Ming dynasty and Greek tragedies [3,4]. Dyadic deaths are relatively unusual with global mortality rates ranging between 0.02 and 0.46 per 1,000,000 with significant national and regional variations [5]. Marzuk et al. were the first to propose a classification based on the relationship between the victim and the perpetrator and labeled the killing of a spouse/intimate partner as uxoricide-suicide/homicide-suicide in a consortial relationship [6,7]. In intimate partner homicide-suicides, perpetrators are usually male [8]. Usually, the perpetrator commits suicide immediately or within a week following the event [7]. This study describes a rare form of dyadic death in which multiple methods were used to kill the intimate partner and then the perpetrator killed himself by hanging after sustaining non-fatal injuries mimicking the victim’s fatal injuries.

## Case presentation

**Figure 1:**
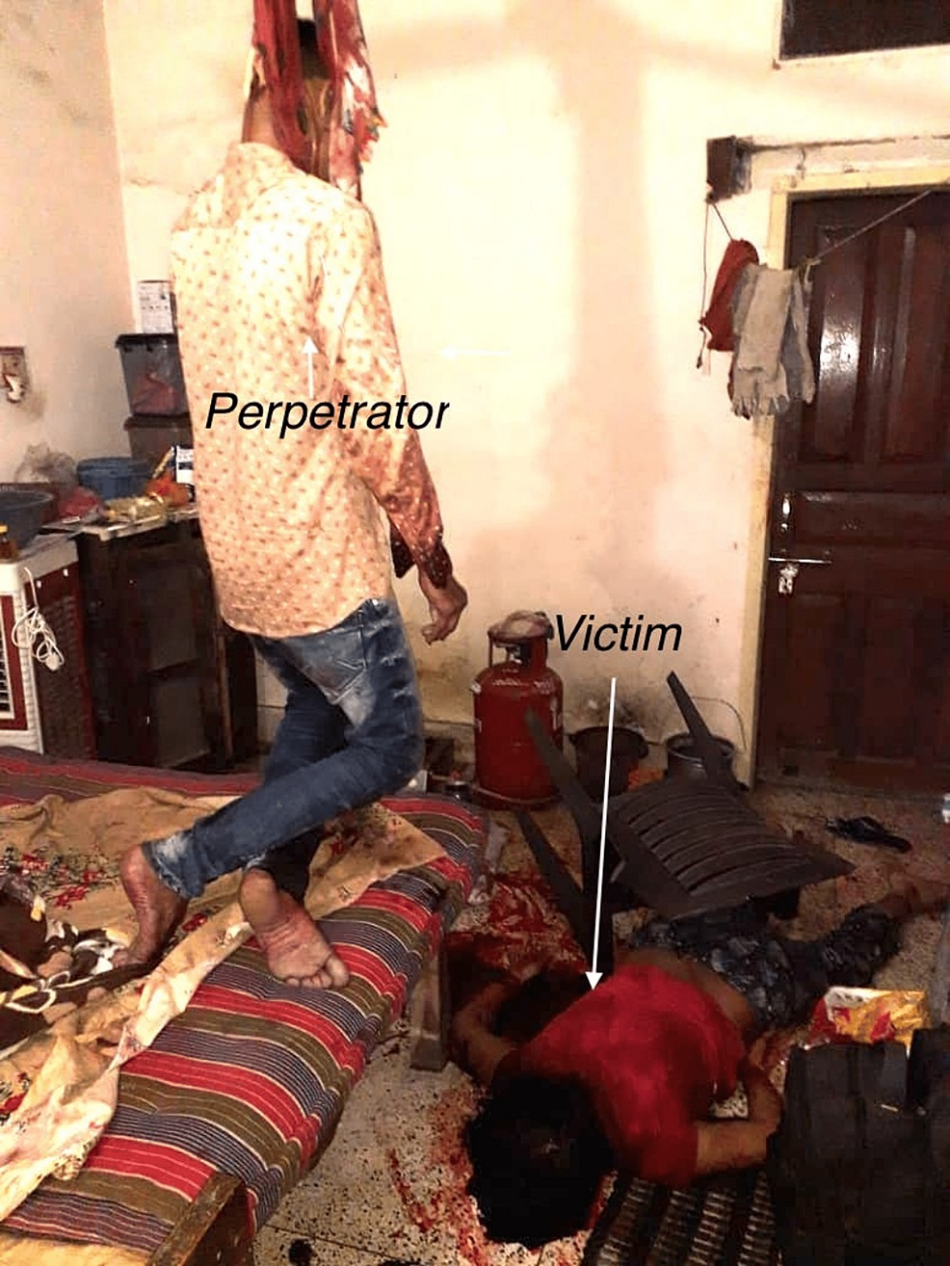
Two bodies at the crime scene. The body of the deceased male was found in a partially hanging state, while the body of the deceased female was lying in a pool of blood.

A couple in a consortial relationship for eight years had an argument and fight one early morning over suspicion of an affair of the female partner. During the argument, she was assaulted by her boyfriend with a hammer on her head and she rushed out of the house seeking help. The neighbors witnessed their heated arguments and saw the boyfriend dragging her inside the house. After some time, the voice of the woman started to fade, and the police were called by the neighbors. On arrival, the police forcibly broke the door and found the dead bodies of the couple. Crime scene findings are depicted in Figure 1. The male was found hanging, while the female partner was lying on the floor in a pool of blood. It was established through the police inquest and statements from friends that the male partner suspected infidelity resulting in frequent quarrels, and, hence, he might have killed his female partner.

Autopsy findings

Victim

**Figure 2:**
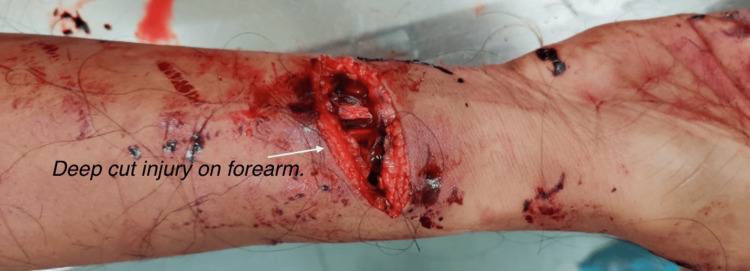
Deep cut injury with complete severance of the underlying radial artery.

The autopsy of the 29-year-old female with a body mass index (BMI) of 23 kg/m^2^ showed blood-soaked clothes, with the face and limbs covered in blood. Incised wounds were present over both forearms. An incised wound over the lower one-third of the right forearm with a slashing injury of the flexor carpi radialis tendon and complete severance of the adjoining radial artery resulted in death (Figure 2).

**Figure 3:**
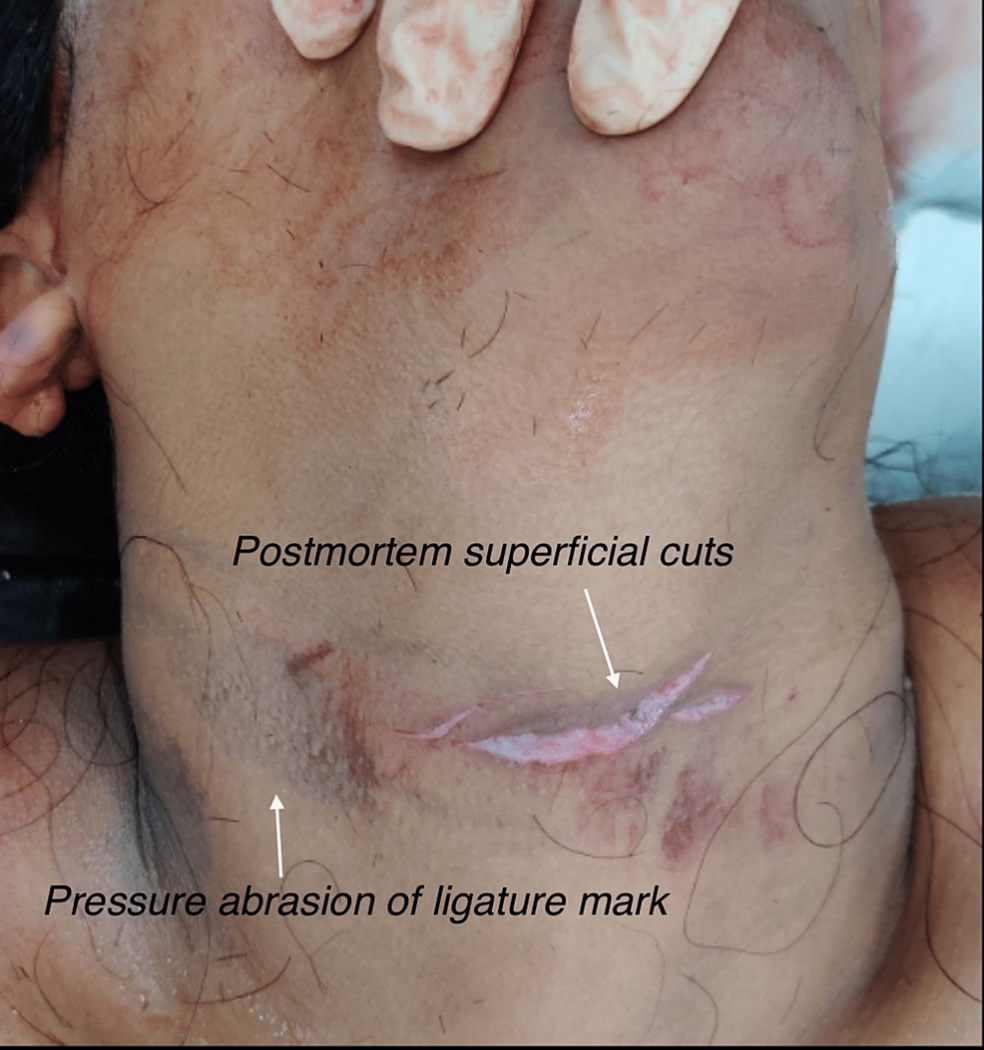
Evidence of a ligature mark on the victim’s neck with a postmortem incised wound.

Pressure abrasion of a ligature mark was noted over the anterolateral aspect of the neck with a maximum width of 2.5 cm. A postmortem superficial incised wound was present on the anterior aspect of the ligature mark with pale margins, and no infiltration of blood or injury was noted on further dissection of the neck (Figure 3).

**Figure 4:**
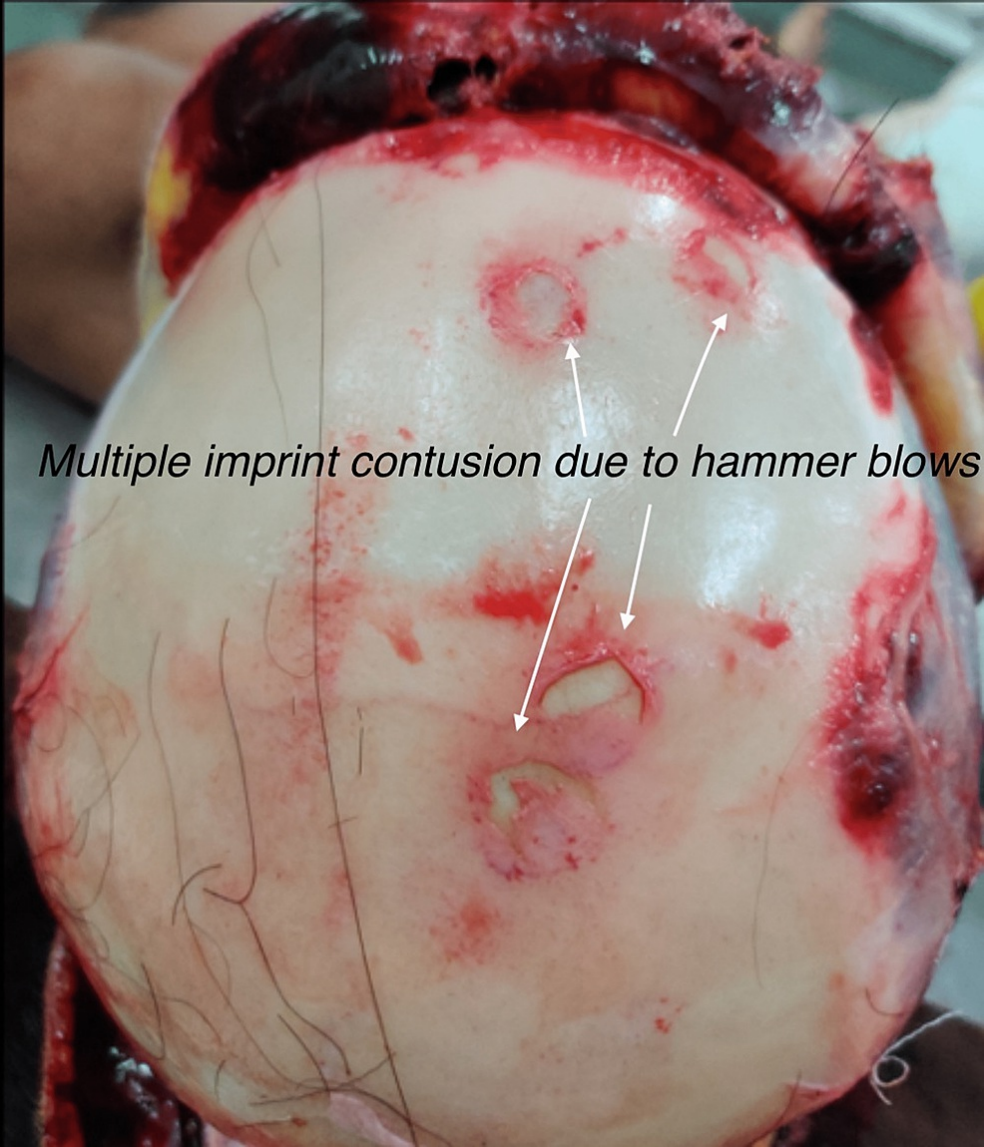
Multiple imprint contusions on the skull vault due to hammer blows.

Multiple split lacerations were present over the scalp, with multiple circular imprint contusions due to hammer blows present on the underlying skull vault without any fracture, intracranial hemorrhage, or injury to the brain (Figure 4).

Internal examination showed pale organs without any signs of asphyxia. The multiplicity of injury indicated aggression, the magnitude of violence, and the determination by the attacker to kill the victim. The cause of death was ascertained as a hemorrhagic shock following a deep wrist injury.

Perpetrator

**Figure 5:**
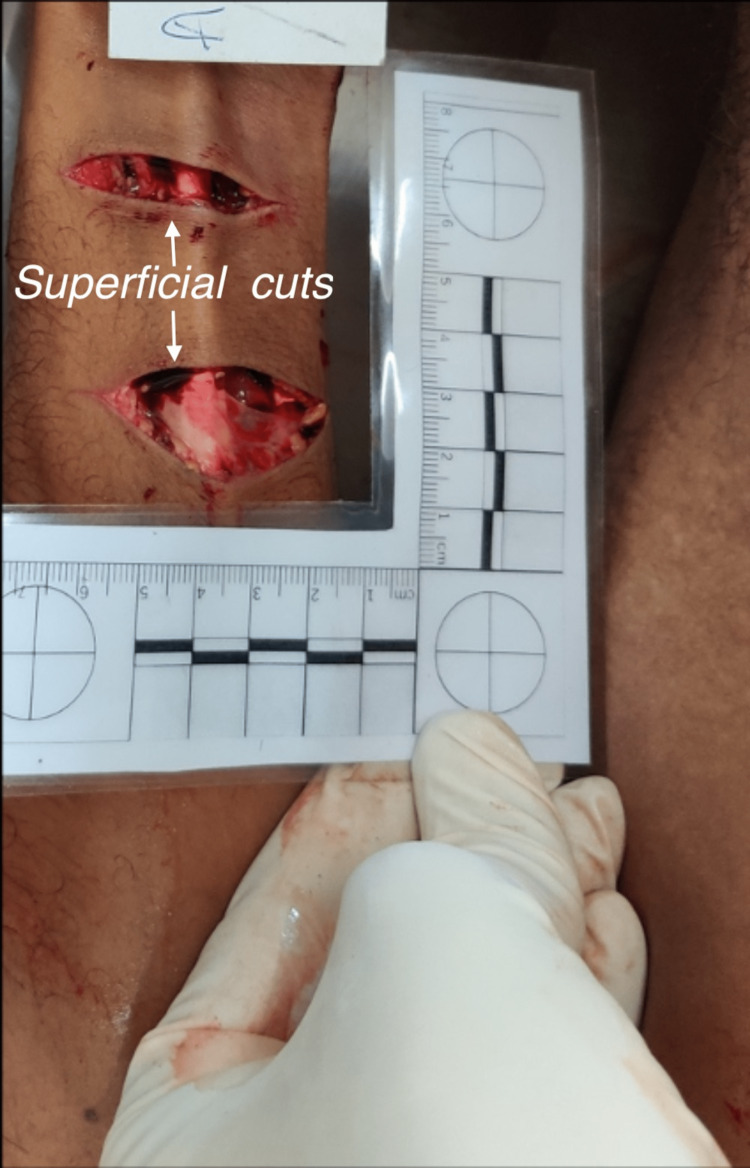
Transverse incised wound on the flexor aspect of the forearm of the perpetrator.

The autopsy of the male perpetrator showed a 28-year-old averagely built and moderately nourished male with a BMI of 24.8 kg/m^2^ and a height of 1.67 m. Clothes were stained with splashes of blood at places. Two parallel incised wounds were present over the left forearm. The wounds were superficial involving the skin and subcutaneous tissue, and the rest of the underlying structures were intact (Figure 5).

**Figure 6:**
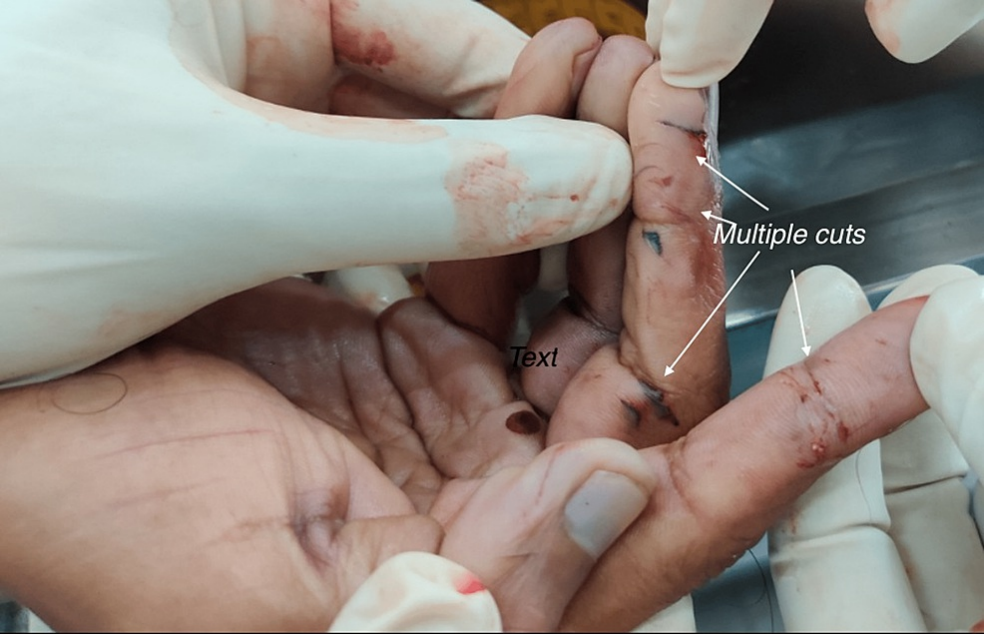
Unintentional self-injury on the perpetrator’s fingers suggestive of the use of a sharp object with bare hands.

Dried blood clots were present on the fingertips and index finger on the right hand. Further examination revealed that the transverse cuts along the longitudinal axis of the fingers were subcutaneously deep, suggesting unintentional self-injury while attempting to attack the victim’s right forearm with a blade (Figure 6).

**Figure 7:**
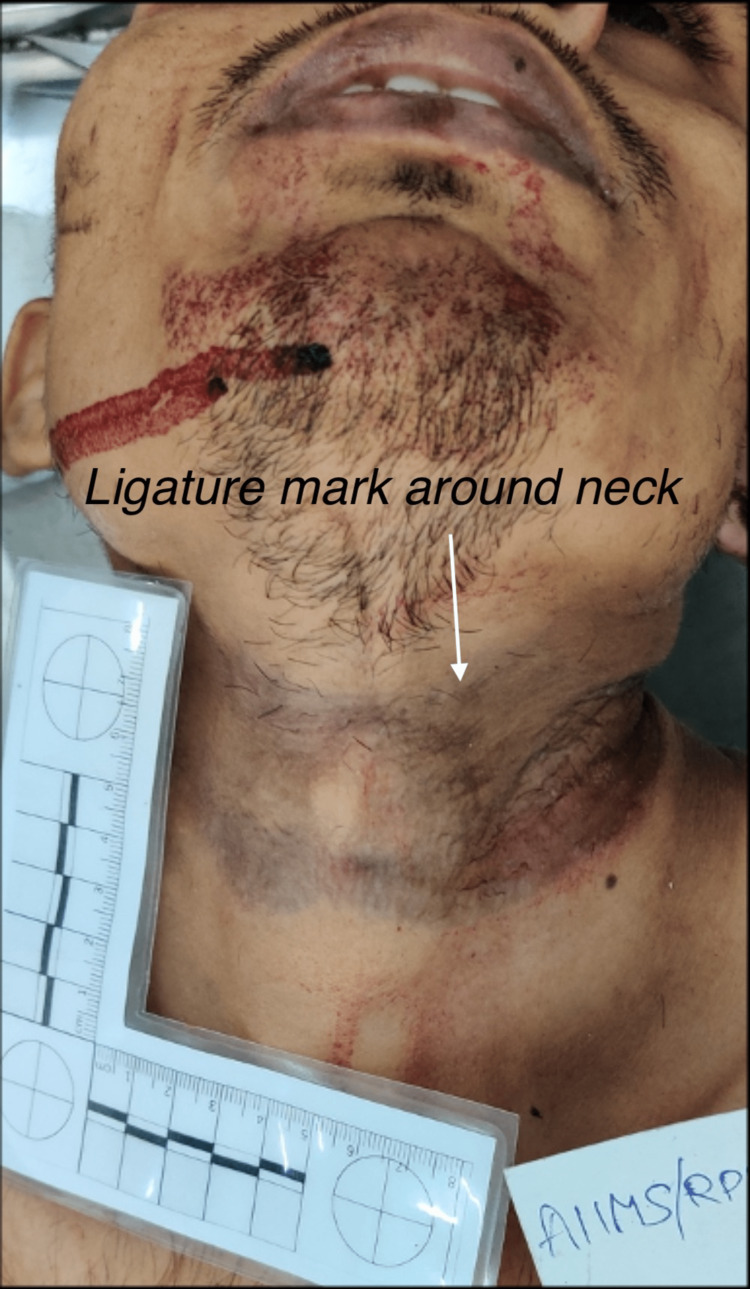
Ligature mark around the neck of the perpetrator.

A blood-stained blade was found in the perpetrator’s jeans pocket. A hanging mark in the form of pressure abrasion was present completely encircling the neck, which was directed obliquely upward and backward with a knot impression over the right occipital region (Figure 7).

No infiltration of blood was noted on the dissection of the neck. Internal examination showed congested organs with evidence of cyanosis and petechial hemorrhages in the lungs. After careful assessment of the history, circumstances of death, exclusion of other causes, and cautious evaluation of the signs described above, the cause of death was ascertained as death due to hanging. The weapon used by the perpetrator to kill the partner was a blade and a hammer, two common household tools found in most Indian homes. Here, we are presenting the cases where multiple methods were used to commit homicide-suicide. This leads to a mirroring of simulating injuries in both the victim as well as the perpetrator.

## Discussion

Suicide committed by perpetrators committing homicide is relatively uncommon and varies from region to region [9]. Stack et al. in their review on homicide-suicide argued persuasively that the closer the ties between the offender and the victim, the more likely the offender is to commit suicide [10]. A male usually commits homicide-suicide after experiencing interpersonal conflict in an intimate relationship, and it usually involves the killing of the female victim [11,12]. A higher than anticipated probability exists that the perpetrator committed the murder-suicide because of an interpersonal conflict arising either due to a lack of communication or a loss of trust. Accordingly, they are also more likely to be angry, hostile, and violent, and to have behaved erratically in the period leading to death, and a similar scenario is reflected in our case report [13-15]. Marzuk et al. classified such a type of homicide-suicide as amorous jealousy and found that in addition to depression, these perpetrators also had histories of abusive relationships with partners [16]. A similar study conducted by Logan et al. in 27 states of America found that perpetrators had a history of domestic violence, were jealous over real or imagined infidelity, and were in the process of breaking up [17]. A study conducted by Santos-Hermoso et al. among perpetrators involved in intimate partner femicide in the prison population of Sweden and Spain found that, as opposed to other criminals, intimate partner aggressors are more specifically perpetrators of femicide and do not exhibit antisocial behavior. Rather, femicide may be committed by an irresponsible partner who lacks impulse control and reacts violently to the conflict [18]. Because firearm possession and use are strictly prohibited in India, household sharp and hard blunt tools are commonly used for killings in violent conflicts and suicides [19,20].

## Conclusions

Two aspects were involved in this case, namely, the perpetrator inflicted non-fatal injuries on his own body that resembled fatal injuries he had inflicted on the victim, but the cause of death differed between intimate partners of the homicide-suicide. The manner of death was discussed based on the meticulous autopsy, crime scene investigation, and other corroborative evidence. In this case report on a homicide-suicide, to confirm the death of the victim, the perpetrator attempted to strangulate the victim with a soft ligature material that created a ligature mark on the victim’s neck, and he also inflicted an incised wound over the ligature to reconfirm it. The perpetrator attempted suicide by inflicting cuts over the forearm and hanged himself. Committing homicide and suicide created a pattern of multiple non-fatal injuries in the perpetrator that simulated fatal injuries in the victim.
